# Identification of Serum microRNA Biomarkers for Tuberculosis Using RNA-seq

**DOI:** 10.1371/journal.pone.0088909

**Published:** 2014-02-20

**Authors:** Hongtai Zhang, Zhaogang Sun, Wenjing Wei, Zhonghui Liu, Joy Fleming, Shuai Zhang, Nan Lin, Ming Wang, Maoshan Chen, Yuhui Xu, Jie Zhou, Chuanyou Li, Lijun Bi, Guangming Zhou

**Affiliations:** 1 Department of Space Radiobiology, Key Laboratory of Heavy Ion Radiation Biology and Medicine, Institute of Modern Physics, Chinese Academy of Sciences, Lanzhou, China; 2 Key Laboratory of Non-coding RNA, Institute of Biophysics, Chinese Academy of Sciences, Beijing, China; 3 University of Chinese Academy of Sciences, Beijing, China; 4 Beijing Key Laboratory of Drug Resistance in Tuberculosis, Beijing Chest Hospital, Capital Medical University, Beijing, China; 5 Huazhong Agriculture University, Hubei, China; 6 College of Life Sciences, Fujian Agriculture and Forestry University, Fuzhou, China; 7 Beijing Genomics Institute, Shenzhen 518083, China; 8 The 4th Peoples’ Hospital, Foshan City, Guangdong Province, China; 9 Beijing Chest Hospital, Capital Medical University, Beijing, China; University of Cape Town, South Africa

## Abstract

Tuberculosis (TB) remains a significant human health issue. More effective biomarkers for use in tuberculosis prevention, diagnosis, and treatment, including markers that can discriminate between healthy individuals and those with latent infection, are urgently needed. To identify a set of such markers, we used Solexa sequencing to examine microRNA expression in the serum of patients with active disease, healthy individuals with latent TB, and those with or without prior BCG inoculation. We identified 24 microRNAs that are up-regulated (2.85–1285.93 fold) and 6 microRNAs that are down-regulated (0.003–0.11 fold) (P<0.05) in patients with active TB relative to the three groups of healthy controls. In addition, 75 microRNAs were up-regulated (2.05–2454.58 fold) and 11 were down-regulated (0.001–0.42 fold) (P<0.05) in latent-TB infected individuals relative to BCG- inoculated individuals. Of interest, 134 microRNAs were differentially-expressed in BCG-inoculated relative to un-inoculated individuals (18 up-regulated 2.9–499.29 fold, 116 down-regulated 0.0002–0.5 fold), providing insights into the effects of BCG inoculation at the microRNA level. Target prediction of differentially-expressed microRNAs by microRNA-Gene Network analysis and analysis of pathways affected suggest that regulation of the host immune system by microRNAs is likely to be one of the main factors in the pathogenesis of tuberculosis. qRT-PCR validation indicated that hsa-miR-196b and hsa-miR-376c have potential as markers for active TB disease. The microRNA differential-expression profiles generated in this study provide a good foundation for the development of markers for TB diagnosis, and for investigations on the role of microRNAs in BCG-inoculated and latent-infected individuals.

## Introduction

Tuberculosis (TB) remains a significant human health issue. It is estimated that up to two billion individuals throughout the world are currently infected with *Mycobacterium tuberculosis* (*M.tb*), 10% of whom will develop active disease during their lifetime [1]. Early diagnosis of TB infection is essential for controlling the spread of the disease. However, accurate diagnosis of TB has been problematic. Sputum smear acid-fast staining is rapid and economic, but has very low sensitivity and specificity. Germiculture requires more than one month and has low sensitivity. Individuals previously inoculated with BCG will produce a positive TB-PPD (purified protein derivative of tuberculin) reaction, reducing the value of the tuberculin skin test [2]. Although QuantiFERON tests, interferon gamma release assays (IGRAs) that test for a positive immune response to *M. tuberculosis*, are not affected by BCG inoculation status, they cannot distinguish between latent TB and active disease. This, in addition to their high cost, has meant that they have not replaced the widely-used tuberculin skin test in China. The development of faster and more accurate clinical diagnostic tools is therefore urgently needed.

Transcriptomic, proteomic and metabolomic profiling, combined with broad scale immunological profiling, has potential to greatly increase our understanding of the pathobiology of TB and provide information helpful in the design of novel intervention strategies [3]. MicroRNAs, a class of small non-coding RNAs approximately 21 nucleotides in length that are found in various organisms [4], are more stable than mRNAs and are thus good candidates for use as biomarkers [5]. They modulate gene function at the post-transcriptional level and act in fine tuning various processes such as development, proliferation, cell signaling, and apoptosis.

The use of microRNAs as potential biomarkers of human disease has been extensively studied and reviewed [6]–[9]. Recently, irregular expression and polymorphisms in the nucleotides of microRNAs that are present in the blood of TB patients have been found to correlate with the initiation and progression of tuberculosis [10]–[12]. Single nucleotide polymorphisms (SNPs) within microRNAs miR-146a and miR-499 (rs2910164 G>C and rs3746444 T>C, respectively) are reported to be related to pulmonary tuberculosis in the Tibetan population, while the C allele at rs3746444 is associated with an increased risk of pulmonary tuberculosis in the Han population [11]. MicroRNA miR-29 acts as an immunological regulator, suppressing IFN-γ production by directly targeting IFN-γ mRNA [12]. Levels of microRNAs in the serum of TB patients and BCG-inoculated individuals have been shown to be significantly different based on microarray-based expression profiling followed by real-time quantitative PCR validation [10]. Wang et al. have also identified some microRNAs that are differentially-expressed in the peripheral blood mononuclear cells (PBMCs) of TB patients and individuals with latent TB infection (LTBI) using similar methods [13].

Here, to generate a broader profile of microRNAs which have potential as biomarkers for distinguishing different disease statuses, we used RNA-seq to identify candidate microRNA biomarkers that are differentially-expressed in the serum of TB patients, individuals with LTBI, and healthy individuals, with or without BCG inoculation. The microRNA differential-expression profiles generated in this study provide a good foundation for the development of markers for TB diagnosis, and for investigations on the role of microRNAs in BCG-inoculated and latent-infected individuals.

## Study Population and Methods

### Ethical Agreement and Consent of Participants

The Ethics committee of the Beijing TB & Thoracic Tumor Research Institute provided ethical clearance. Participation in the investigation from March 2005 to April 2008 was voluntary and each participant’s informed written consent was obtained. Characteristics of the study population are given in Table 1.

**Table 1:** Demographic characteristics of the study population.

	Active TB	LTBI	BCG-inoculated controls	Un-inoculated controls
Participants	15	14	22	46
Gender				
Female	5/15	10/14	10/22	25/46
Male	10/15	4/14	12/22	21/46
Age (years; mean±SEM)	35.22±6.19	31.41±4.56	36.22±9.24	28.23±8.36
Tuberculin skin test				
Positive (>10 mm)	15/15	13/14	20/22	
Positive (5–10 mm)		1/14	2/22	
Negative (0 mm)				46/46

LTBI, individuals with latent TB infection.

### Serum Collection

Five milliliters of venous blood was collected from each participant in the morning (on an empty stomach). To harvest cell-free serum, blood was drawn into a sterile polyolefin resin tube without anticoagulant. After standing for 20 min at room temperature, samples were centrifuged at 3,000 rpm for 10 min, and the supernatant serum was quickly removed, aliquoted, and stored immediately in liquid nitrogen.

### RNA Isolation

Serum samples in each group were pooled and RNA was isolated using a miRNeasy RNA isolation kit (Qiagen) according to the manufacturer’s protocol, except that 400 µl of serum was denatured initially with 10 volumes of Qiazol reagent. RNA was eluted with 105 µl distilled water to yield 80 µl of RNA elutant [14].

### Solexa Sequencing

Briefly, after PAGE purification, a pair of Solexa adaptors were ligated to the 5′ and 3′ ends of small RNA molecules <30 nt in length. These small RNA molecules were then amplified for 17 cycles using adaptor primers, and fragments of around 90 bp (small RNA+adaptors) were isolated after electrophoresis on an agarose gel. Purified DNA was used directly for cluster generation and sequencing analysis using an Illumina-Solexa Sequencer according to the manufacturer’s instructions. Image files generated by the sequencer were processed to produce digital-quality data. Clean reads were compared with miRBase (release 10.0) and the total copy number of each sample was normalized to 100 000.

### Statistical Analysis

Determination of differentially expressed miRNAs. Fold changes in expression (sample group versus control) were calculated for each gene and miRNA as log_2_ ratios using normalized TPM (transcripts per million reads) values according to the formula: Fold-change = log2 (sample group/control). Rigorous significance tests, as described by Audic and Claverie (1997) [15], were then performed to determine differentially expressed miRNAs. Differences between samples were regarded as significant at P<0.05.

### Pathway Enrichment Analysis

A list of genes predicted to be targeted by the differentially-expressed miRNAs was obtained using TargetScan 6.0 (http://www.targetscan.org). Predicted miRNA target genes were analyzed for enrichment in KEGG pathways using DAVID (http://david.abcc.ncifcrf.gov) with default settings.

### Validation of miRNAs Targeting NFAT5

We overexpressed three miRNAs predicted to target NFAT5 (*hsa-miR-376c, hsa-miR-516b* and *hsa-miR-486-5p*) in HEK293 cells and analysed their transcript levels. *Cel-miR-67-3p* was used as a negative control, and 18s rRNA was used as an endogenous control. HEK293 cells were transfected respectively with these four miRNAs mimics (RIBOBIO, PRC) using Lipofectamine 2000 (Invitrogen, USA), then incubated at 37°C (5% CO_2_) for 24 h before extracting total RNA using TRIzol reagent (Invitrogen, Carlsbad, CA, USA) according to the manufacturer’s protocol.

Real-Time qPCR was performed using TransScript Green Two-Step qRT-PCR SuperMix (Transgen, PRC). 500 ng of total RNA was converted to cDNA with specific reverse qPCR primers according to the manufacturer’s protocol. qPCR was performed in a total reaction volume of 20 µl, including 10 µl of 2×*TransStart* Top Green qPCRSuperMix, 0.8 µl of qPCR Primers (5 µM), 1 µl of First-Strand cDNA (18s cDNA was diluted 1∶1000) and 8.2 µl double-distilled water. Reactions were performed and analyzed using a CFX96 Real-Time PCR Detection System (Bio-Rad, USA). PCR primers for 18s rRNA were 5′-GTAACCCGTTGAACCCCATT-3′ (forward) and 5′-CC ATCCAATCGGTAGTAGCG-3′ (reverse), and those for NFAT5 were 5′-GGCATTCAAAATAACTGTAGTCAGC-3′ (forward) and 5′-AAGTGTTGT CACAGGTGGCT-3′ (reverse). qPCR reactions were performed for each cDNA in triplicate and the whole experiment was repeated three times. Cycling parameters for qPCR were as follows: (1) an initial denaturation step of 30 s at 94°C; (2) 35 cycles of 5 s at 95°C, and 30 s at 60°C. The relative quantity (RQ) of mRNAs was determined by 2^−ΔΔCT^, where ΔCT = (CT_NFAT5_–CT _endogenous control 18s_) and ΔΔCT = (ΔCT−average ΔCT of all the samples).

### Real-time Quantification of Serum microRNAs

Reverse transcription reactions were performed using a TaqMan miRNA Reverse Transcription kit (Ambion, TN, USA) and miRNA-specific stem-loop primers according to the manufacturer’s instructions. Each reaction mixture for real-time quantitative PCR contained 2.5 µl 2X TaqMan Universal PCR Master Mix without AmpErase UNG, 0.25 µl miRNA-specific primer/probe mix, and 2.25 µl diluted RT product (1∶15) in a total volume of 5 µl. Reactions were amplified using the following thermal cycling parameters: 95°C for 10 min, followed by 40 cycles of 95°C for 15 s, and 60°C for 1 min, followed by holding at 4°C. Raw data were analyzed with SDS Relative Quantification Software version 2.2.3 (Applied BioSystems, Inc.), generally using the automatic cycle threshold (Ct) setting for assigning the baseline and threshold for Ct determination. Each sample was normalized using spiked-in synthetic *C. elegans* miRNAs as controls. Experiments were performed in triplicate.

## Results

### Study Population

A total of 97 volunteers took part in this study. Fifteen active pulmonary tuberculosis patients were recruited from the Beijing Chest Hospital. Eighty-two healthy volunteers from the Beijing Chest Hospital and the Beijing General Team of the Armed Police Forces were divided into three groups (**Table 1**) based on TB-PPD tuberculin skin test results and whether they had previously been inoculated with BCG. Group A (latent TB infected group) consisted of individuals with a positive TB-PPD Tuberculin skin test who had not been BCG-inoculated; Group B (inoculated group) consisted of individuals with a positive TB-PPD TST who had been BCG-inoculated; and Group C (healthy controls) consisted of individuals with a negative TB-PPD Tuberculin skin test who had not been BCG-inoculated.

### Analysis of Differentially-expressed Serum microRNAs

**Figure 1:**
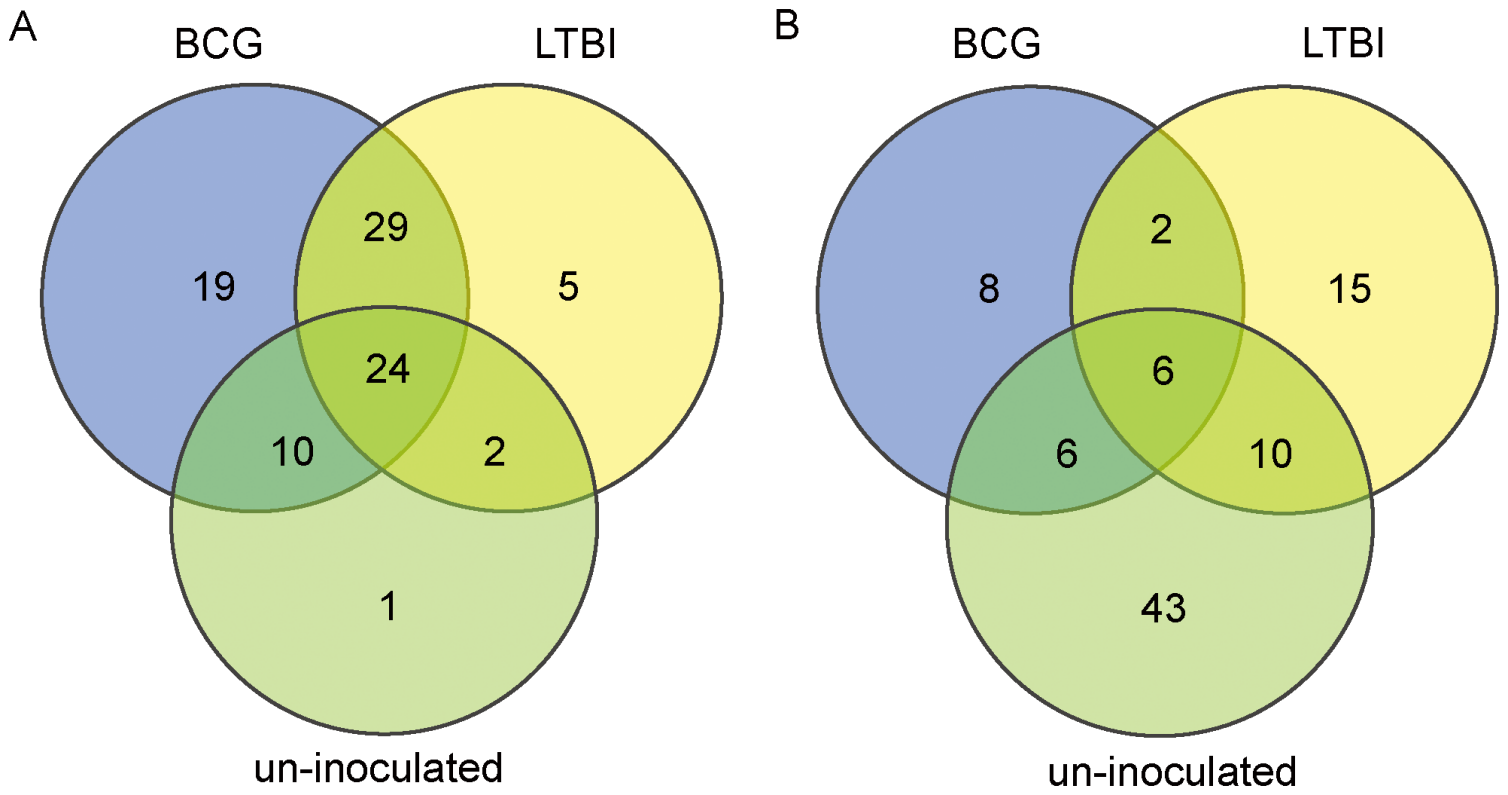
Venn diagram illustrating the distribution of microRNAs which showed significantly altered expression in TB patients compared with three control groups (LTBI, BCG-inoculated, and un-inoculated individuals). A, microRNAs which were up-regulated in serum from TB patients compared with the control groups; B, microRNAs which were down-regulated in serum from TB patients compared with the control groups. BCG, BCG-inoculated; LTBI, individuals with latent TB infection; Healthy, un-inoculated controls.

Serum microRNAs were sequenced using Solexa sequencing to determine the serum microRNA profiles of patients with active pulmonary tuberculosis, individuals with latent TB infection, healthy BCG-inoculated and un-inoculated individuals. A total of 904 microRNAs were obtained, 162 of which showed significantly altered expression in the active TB group compared with the three control groups (LTBI, and healthy BCG-inoculated, and un-inoculated individuals) (**Figure 1**). The expression of 24 microRNAs was up-regulated 2.85–1285.93 fold in all three control groups compared to TB patients, while 6 microRNAs were down-regulated 0.003–0.11 fold (P<0.05) (**Table S1**). In addition, expression of 60 microRNAs was up-regulated 2.10–1285.93 fold in serum from TB patients compared to LTBI individuals, while 33 microRNAs were down-regulated 0.0012–0.4896 fold (P<0.05) (**Table S2**). Expression of 82 microRNAs was up-regulated 2.10–6340.96 fold in serum from TB patients compared with BCG-inoculated individuals (group B), while 22 microRNAs were down-regulated 0.003–0.1106 fold (P<0.05) (**Table S3**) and the expression of 37 microRNAs was up-regulated 2.19–6340.96 fold in serum from TB patients compared to un-inoculated individuals, while 65 microRNAs were down-regulated 0.0002–0.4880 fold (P<0.05) (**Table S4**).

Comparison of microRNAs differentially-expressed in LTBI and BCG-inoculated individuals showed that 83 microRNAs were up-regulated 1.1–2454.58 fold and 11 were down-regulated 0.001–0.418 (P<0.05) (**Table S5**). 138 microRNAs were differentially-expressed in LTBI and un-inoculated individuals, of which 44 were up-regulated 2.52–843.76 fold and 94 were down-regulated 0.0006–0.4474 fold (**Table S6**), while 134 were differentially expressed in inoculated and un-inoculated individuals, of which 18 were up-regulated 2.69–499.29 fold and 116 were down-regulated 0.0003–0.5049 fold (**Table S7**).

### Pathway Enrichment Analysis

**Figure 2:**
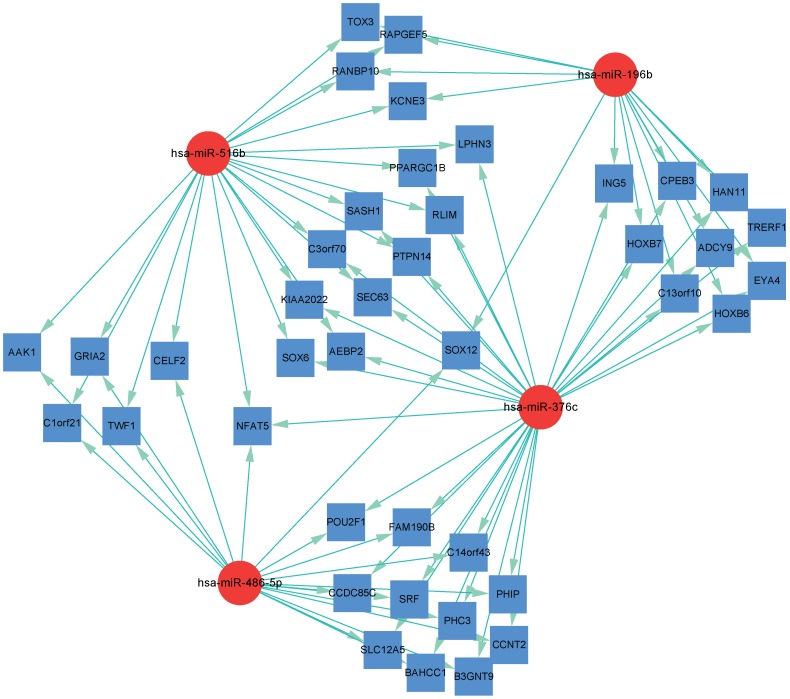
microRNA-gene network for hsa-miR-196b, hsa-miR-516b, hsa-miR-376c and hsa-miR-486-5p. The microRNA-gene network was built using gene expression data and predicted interactions from the TargetScan and PicTar microRNA databases. Red circles represent microRNAs and blue squares represent genes; their relationship is represented by the edges.

**Figure 3:**
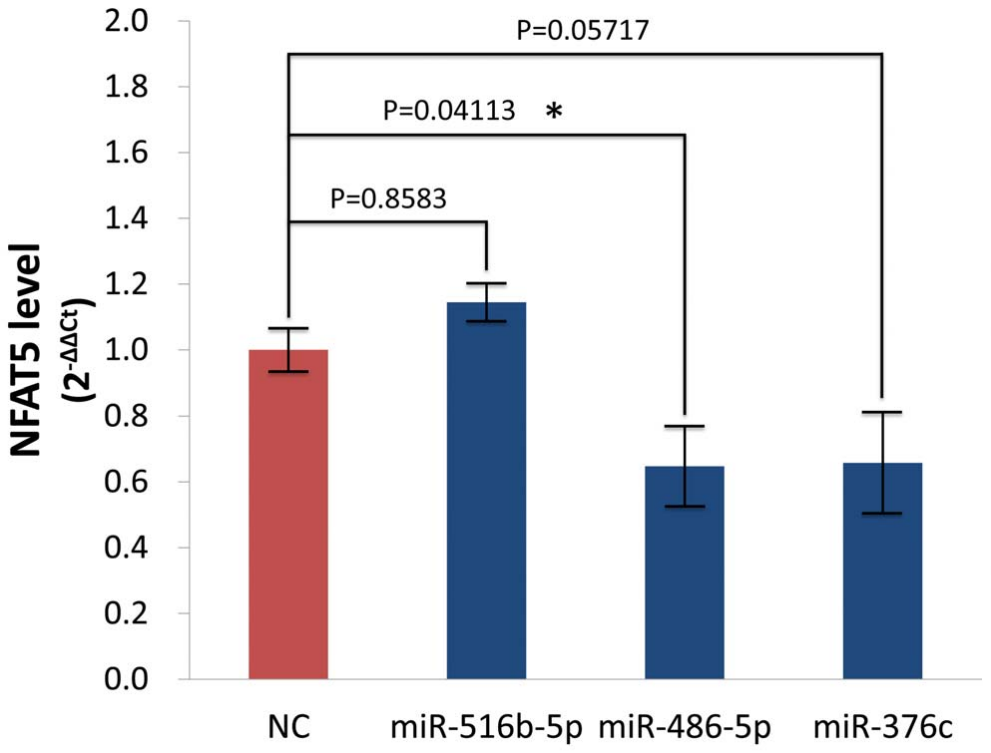
Effects of miRNA overexpression on NFAT5 mRNA levels (2^−ΔΔCT^) in HEK293 cells. HEK293 cells were transfected respectively with four miRNA mimics (*hsa-miR-376c*, *hsa-miR-516b*, *hsa-miR-486-5p* and *cel-miR-67-3p*) and incubated at 37°C (5% CO_2_) for 24 h. NFAT5 mRNA levels were measured in extracts of total RNA using RT-qPCR. NC: negative control (*cel-miR-67-3p*). Data presented are mean values from three independent experiments, n = 3. Error bars show the s.e.m. The significance of comparisons was tested using the Student’s *t*-test; *: P<0.05.

In an attempt to learn more about the differentially-expressed microRNAs, we used TargetScan to analyze their target genes and DAVID to identify biochemical pathways in which conserved targets are involved. Figure 2 shows the microRNA-Gene-Network for miRNAshsa-miR-516b,hsa-miR-196b, hsa-miR-376c and hsa-miR-486-5p. These four miRNAs are predicted by TargetScan and PicTar to have 42 targets. Some miRNAs were predicted to target the same mRNA, suggesting that they may be involved in the same pathways during tuberculosis disease progression. For example, both hsa-miR-376c and hsa-miR-516b had 11 targets in common, while hsa-miR-376c and hsa-miR-486-5p had 12 targets in common. In addition, hsa-miR-196b and hsa-miR-376c shared the same eight target mRNAs, while hsa-miR-376c, hsa-miR-516b and hsa-miR-486-5p all targeted NFAT5 whose gene and protein expression are known to be strongly induced by *M. tuberculosis* [16]. To provide some validation of these predictions, we overexpressed the three miRNAs which target NFAT5 in HEK293 cells and measured NFAT5 mRNA levels using real-time qPCR (Figure 3). While NFAT5 mRNA levels were not significantly affected by overexpression of *hsa-miR-516b*, overexpression of *hsa-miR-376c* or *has-miR-486-5p* did result in down-regulation of NFAT5, with *has-miR-486-5p* having the most significant effect (P = 0.0411). These results suggest that *hsa-miR-376c* and *has-miR-486-5p* may be involved in regulating the NFAT5 pathway, possibly leading to the higher levels of NFAT5 expression observed in patients with active TB [16].

Pathways involved in cell growth, movement and multiplication, such as focal adhesion, MAPK signaling, insulin signaling, chronic myeloid leukemia and TGF-beta signaling were significantly enriched in the differentially-expressed microRNAs in this study (**Table S8**). Of note, B cell receptor signaling pathways and T cell receptor signaling pathways were also significantly enriched. These results suggest that these microRNAs mainly affect the immune system in individuals infected with TB.

### Validation of microRNA Expression Using qRT-PCR

**Figure 4:**
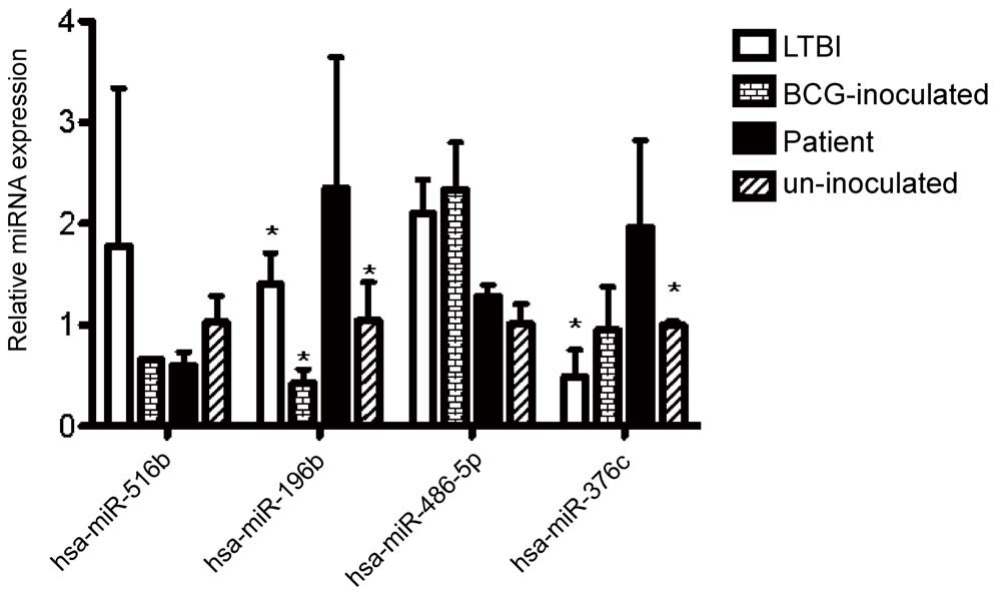
Validation of microRNA expression by qRT-PCR. qRT-PCR analysis confirms Solexa sequencing data. Data, normalized to *cel-miR-238*, are presented as means ±SD (n = 10). *Hsa-miR-196b* and *hsa-miR-376c* were up-regulated in TB patients in comparison with each of the other three groups. In LTBI individuals *hsa-miR-516b* was up-regulated, while *hsa-miR-376c* was down-regulated. In BCG-inoculated individuals *hsa-miR-486-5p* was up-regulated, while *hsa-miR-196b* was down-regulated.

Results from Solexa sequencing suggested that hsa-miR-516b, hsa-miR-196b and hsa-miR-376c are significantly up-regulated in patients with active TB, while hsa-miR-486-5p is significantly down-regulated. To validate these significant differential changes in expression, we performed qRT-PCR. MicroRNA samples were pooled from 10 individuals per group to rule out the possibility that differences in gene expression were due to variation in the relative abundance of cell populations between individuals. Expression levels of each of the four microRNAs were then determined using qRT-PCR. Although results for hsa-miR-486-5p and hsa-miR-516b were not consistent with those from RNA-Seq (**Figure 4**), hsa-miR-196b was consistently significantly up-regulated in serum from patients with active TB compared with the other three groups using both techniques, and expression of hsa-miR-376c was significantly higher in serum from TB patients than in serum from individuals with LTBI and un-inoculated healthy individuals, suggesting that these two microRNAs have potential as diagnostic markers.

## Discussion

Routine clinical methods for diagnosing TB, including radiography, sputum culture, the tuberculin skin test (TST) and QuantiFERON IGRA tests, have many shortcomings. Finding new biomarkers for tuberculosis is not only necessary for diagnosing patients with TB, but also for the staging or classification of TB, TB prognosis, and TB drug and vaccine trials [3], [17]. The use of miRNAs as biomarkers for different kinds of cancers has been intensively investigated and some promising candidates have been developed [18]. Here, we have used RNA-seq and qRT-PCR to identify microRNAs involved in tuberculosis which have potential as biomarkers for this infectious disease.

Over the past decade, a number of different approaches for quantifying microRNAs have been described, including cDNA arrays [19]–[22], a modified Invader assay [23] and real-time PCR measurement of miRNA precursors [24], [25]. RNA-seq is a more effective method for identifying significant changes in the levels of microRNAs between healthy people and individuals with active disease and for identifying microRNAs with potential as biomarkers for TB diagnosis. Here, we identified and quantified the expression of a total of 904 microRNAs in serum from four experimental groups, namely individuals with active TB, individuals with latent TB infections, and healthy individuals, with or without prior BCG inoculation.

Identification of biomarkers which can discriminate between latent TB infection and active TB disease is one of the most important issues in TB prevention and control [26], [27]. Recently, several studies on differences in the levels of microRNAs between patients with active TB and individuals with latent TB infection have been reported [13], [14], [28]. Here, we have tried to identify further microRNAs that are specific and can be used to discriminate between the four experimental groups. We identified 24 microRNAs that are up-regulated and 6 that are down-regulated in active TB patients compared with the three groups of healthy controls. Our results confirm the potential of hsa-miR-29a [10], [13], and hsa-miR-22 [13] as biomarkers in TB diagnosis. We have also identified other microRNAs which can discriminate between active TB patients and latently-infected individuals; 59 miRNAs were down-regulated and 33 miRNAs were up-regulated in patients with active disease. In addition to the greater sensitivity of RNA-Seq for the detection of microRNAs, the inclusion of three control groups (latent TB, BCG-inoculated and un-inoculated groups) in this study increases the accuracy of identifying markers that are genuinely associated with active disease.

BCG inoculation is routinely performed on new-born babies throughout most regions in China. The development of an easy method for discriminating between BCG-inoculated individuals and those with latent TB infections is thus of great importance. To that end, we included the detection of microRNA in the serum of BCG-inoculated individuals in our study. Results showed that 83 microRNAs were up-regulated and 11 were down-regulated in LTBI individuals in comparison with BCG-inoculated individuals. We provide evidence here for the first time that a significant change in the levels of microRNAs occurs in the serum of latent TB infected individuals relative to BCG-inoculated individuals.

Many differentially-expressed miRNAs, such as the four microRNAs validated here using qRT-PCR, are involved in inflammation. Hsa-miR-516b, hsa-miR-486-5p and hsa-miR-376c all target NFAT5 (nuclear factor of activated T-cells 5) (http://ferrolab.dmi.unict.it/index.html). Hsa-miR-486-5p is associated with cancers, such as lung cancer [29], [30], gastric cancer [31],and neuroblastoma [32]. Hsa-mir-196b, first reported in 2004 [33], inhibits proliferation and induces apoptosis in endometriotic stromal cells [34] and is associated with cervical tumours [35]. Hsa-miR-376c, first reported by Sylvius et al [36] is associated with muscular dystrophy [33]. We hypothesize that the differential expression of these microRNAs in patients with active TB, individuals with LTBI, and BCG-inoculated and un-inoculated healthy controls results in differences in expression of their target mRNAs, however, this requires further experimental verification.

Of the targets predicted here, NFAT5, a member of the nuclear factors of activated T cells (NFAT) family of transcription factors and a component of the mitogen-activated protein kinase (MAPK) pathway, is of particular interest as it has previously been linked with TB; the innate immune response to *M.tb* infection strongly induces NFAT5 gene and protein expression [16]. In addition, other proteins belonging to the NFAT family are known to play a central role in inducible gene transcription during the immune response [37]. NFAT5 expression has been shown to depend on p38 mitogen-activated protein kinase (MAPK) [38], [39]; addition of a p38 MAPK inhibitor was found to correlate with decreased NFAT5 expression, even in the presence of osmotic stress signals. NFAT5 has also been found to play a crucial role in *M.tb* regulation of HIV-1 replication on co-infection via a direct interaction with the viral promoter. These findings suggest a general role for NFAT5 in *M.tb*-mediated control of gene expression.

In conclusion, this is the first comprehensive RNA-seq study of global microRNA expression levels in different individuals according to their TB disease and inoculation status. We have been able to accurately identify microRNAs that are significantly up- or down-regulated in different groups according to their TB disease and inoculation status. These results provide an excellent starting point for further studies regarding the potential of these microRNAs as biomarkers for diagnosis and prognosis.

### NCBI Short Read Archive Accession Numbers

Raw sequencing data are available under Accession Numbers XQA(SRR1025184), XQB(SRR1025185), XQC(SRR1025186) and XQD(SRR1025187).

## Supporting Information

Table S1 **Fold changes in the expression of the microRNAs in serum from patients with active TB compared with the other controls (LTBI, BCG-inoculated and un-inoculated individuals).**
(DOC)

Table S2 **Fold changes in the expression of microRNAs in serum from patients with active TB compared with LTBI.**
(DOC)

Table S3 **Fold changes in the expression of microRNAs in serum from patients with active TB compared with BCG-inoculated individuals.**
(DOC)

Table S4 **Fold changes in the expression of microRNAs in serum from patients with active TB compared with BCG un-inoculated individuals.**
(DOC)

Table S5 **Fold changes in the expression of microRNAs in serum from individuals with LTBI compared with BCG-inoculated individuals.**
(DOC)

Table S6 **Fold changes in the expression of microRNAs in serum from individuals with LTBI compared with BCG un-inoculated individuals.**
(DOC)

Table S7 **Fold changes in the expression of microRNAs in serum from BCG-inoculated compared with BCG un-inoculated individuals.**
(DOC)

Table S8 **Pathways represented among the genes predicted to be targeted by microRNAs differentially-expressed in serum from patients with active TB compared with the other controls (LTBI, BCG-inoculated and un-inoculated individuals).**
(DOC)
