# Supplementary material for: Identification of Serum microRNA Biomarkers for Tuberculosis Using RNA-seq
Source: PLoS One. 2014 Feb 20;9(2):e88909. doi: 10.1371/journal.pone.0088909 (PMC3930592; doi:10.1371/journal.pone.0088909)
Supplement: Table S1 — Fold changes in the expression of the microRNAs in serum from patients with active TB compared with the other controls (LTBI, BCG-inoculated and un-inoculated individuals). (DOC) [file pone.0088909.s001.doc]

**Table S1 Fold changes in the expression of microRNAs in serum from patients with active TB compared with the other controls (LTBI, BCG-inoculated and un-inoculated individuals)**

| Up-regulated microRNAs | Fold change | Down-regulated microRNAs | Fold change |
| --- | --- | --- | --- |
| hsa-miR-30a | 5.23 | hsa-miR-16 | 0.0003 |
| hsa-miR-516b | 22.49 | has-miR-29b | 0.0017 |
| hsa-miR-199a-5p | 20.13 | hsa-miR-451 | 0.0009 |
| hsa-miR-23a | 5.4 | has-miR-889 | 0.0034 |
| hsa-miR-195 | 4.1 | hsa-miR-486-5p | 0.1106 |
| hsa-miR-99b | 9.57 | hsa-miR-503 | 0.0021 |
| hsa-miR-22 | 5.71 |  |  |
| hsa-miR-196b | 1285.93 |  |  |
| hsa-miR-10a | 4.45 |  |  |
| hsa-miR-206 | 5.01 |  |  |
| hsa-miR-145 | 19.91 |  |  |
| has-miR-29a | 2.85 |  |  |
| hsa-miR-10b | 7.3 |  |  |
| hsa-miR-204 | 487.77 |  |  |
| hsa-miR-376c | 487.77 |  |  |
| hsa-miR-202* | 5.02 |  |  |
| hsa-miR-127-3p | 8.11 |  |  |
| hsa-miR-1283 | 399.08 |  |  |
| hsa-miR-151-5p | 19.29 |  |  |
| hsa-miR-194 | 7.98 |  |  |
| hsa-miR-200a | 6.59 |  |  |
| hsa-miR-203 | 7.23 |  |  |
| hsa-miR-296-5p | 399.08 |  |  |
| hsa-miR-30c | 11.48 |  |  |
| hsa-miR-520d-5p | 399.08 |  |  |
