# Supplementary material for: Identification of Serum microRNA Biomarkers for Tuberculosis Using RNA-seq
Source: PLoS One. 2014 Feb 20;9(2):e88909. doi: 10.1371/journal.pone.0088909 (PMC3930592; doi:10.1371/journal.pone.0088909)
Supplement: Table S2 — Fold changes in the expression of microRNAs in serum from patients with active TB compared with LTBI. (DOC) [file pone.0088909.s002.doc]

**Table S2 Fold changes in the expression of microRNAs in serum from patients with active TB compared with LTBI.**

| Up-regulated microRNAs | Fold change | Down-regulated microRNAs | Fold change |
| --- | --- | --- | --- |
| hsa-let-7a | 2.4 | hsa-miR-130b | 0.0021 |
| hsa-let-7c | 2.53 | hsa-miR-142-5p | 0.0028 |
| hsa-let-7d | 3 | hsa-miR-143* | 0.002 |
| hsa-let-7e | 3.9 | hsa-miR-144 | 0.0036 |
| hsa-let-7g | 2.25 | hsa-miR-148a | 0.0019 |
| hsa-miR-101 | 2.32 | hsa-miR-15a | 0.0015 |
| hsa-miR-103 | 2.39 | hsa-miR-16 | 0.0004 |
| hsa-miR-107 | 3.42 | hsa-miR-185 | 0.2541 |
| hsa-miR-10a | 3.82 | hsa-miR-18a | 0.0036 |
| hsa-miR-10b | 21.39 | hsa-miR-23b* | 0.002 |
| hsa-miR-122 | 5.12 | hsa-miR-30a* | 0.0023 |
| hsa-miR-125b | 798.16 | hsa-miR-375 | 0.0036 |
| hsa-miR-127-3p | 443.42 | hsa-miR-411 | 0.0016 |
| hsa-miR-1283 | 399.08 | hsa-miR-423-5p | 0.4868 |
| hsa-miR-1307 | 354.74 | hsa-miR-451 | 0.0014 |
| hsa-miR-145 | 1241.59 | hsa-miR-483-5p | 0.0015 |
| hsa-miR-151-5p | 310.4 | hsa-miR-486-5p | 0.2335 |
| hsa-miR-192 | 6.23 | hsa-miR-487b | 0.003 |
| hsa-miR-194 | 620.79 | hsa-miR-503 | 0.0021 |
| hsa-miR-195 | 2.64 | hsa-miR-598 | 0.0019 |
| hsa-miR-196b | 1285.93 | hsa-miR-675 | 0.002 |
| hsa-miR-1974 | 665.14 | hsa-miR-889 | 0.0036 |
| hsa-miR-199a-3p | 2.1 | hsa-miR-92a | 0.4896 |
| hsa-miR-199a-5p | 6.76 | hsa-miR-92b* | 0.0019 |
| hsa-miR-199b-3p | 2.1 | hsa-miR-93 | 0.1156 |
| hsa-miR-200a | 665.14 | hsa-let-7e* | 0.0049 |
| hsa-miR-200c | 487.77 | hsa-miR-155 | 0.0039 |
| hsa-miR-202* | 5.7 | hsa-miR-342-3p | 0.1239 |
| hsa-miR-203 | 17.34 | hsa-miR-517a | 0.0039 |
| hsa-miR-204 | 487.77 | hsa-miR-517b | 0.0039 |
| hsa-miR-206 | 3.47 | hsa-miR-543 | 0.0049 |
| hsa-miR-21 | 2.58 | hsa-miR-625 | 0.0039 |
| hsa-miR-215 | 798.16 | hsa-miR-29b | 0.0012 |
| hsa-miR-22 | 3.54 |  |  |
| hsa-miR-221 | 4.14 |  |  |
| hsa-miR-23a | 4.42 |  |  |
| hsa-miR-24 | 3.16 |  |  |
| hsa-miR-26a | 5.17 |  |  |
| hsa-miR-27b | 0.27 |  |  |
| hsa-miR-296-5p | 399.08 |  |  |
| hsa-miR-29c | 665.14 |  |  |
| hsa-miR-30a | 4.5 |  |  |
| hsa-miR-30c-1* | 443.42 |  |  |
| hsa-miR-31 | 487.77 |  |  |
| hsa-miR-320a | 2.2 |  |  |
| hsa-miR-320b | 2.28 |  |  |
| hsa-miR-340 | 7.23 |  |  |
| hsa-miR-34c-5p | 2.1 |  |  |
| hsa-miR-376c | 487.77 |  |  |
| hsa-miR-378 | 4.16 |  |  |
| hsa-miR-424 | 12.57 |  |  |
| hsa-miR-432 | 2.89 |  |  |
| hsa-miR-516b | 7.52 |  |  |
| hsa-miR-520d-5p | 399.08 |  |  |
| hsa-miR-744 | 3.25 |  |  |
| hsa-miR-9 | 221.71 |  |  |
| hsa-miR-99a | 9.59 |  |  |
| hsa-miR-99b | 5.02 |  |  |
| hsa-miR-30c | 3.9 |  |  |
| hsa-miR-433 | 133.03 |  |  |
