# Supplementary material for: Identification of Serum microRNA Biomarkers for Tuberculosis Using RNA-seq
Source: PLoS One. 2014 Feb 20;9(2):e88909. doi: 10.1371/journal.pone.0088909 (PMC3930592; doi:10.1371/journal.pone.0088909)
Supplement: Table S3 — Fold changes in the expression of microRNAs in serum from patients with active TB compared with BCG-inoculated individuals. (DOC) [file pone.0088909.s003.doc]

**Table S3 Fold changes in the expression of microRNAs in serum from patients with active TB compared with BCG-inoculated individuals**

| Up-regulated microRNAs | Fold change | Down-regulated microRNAs | Fold change |
| --- | --- | --- | --- |
| hsa-let-7a | 5.76 | hsa-miR-139-3p | 0.004 |
| hsa-let-7b | 3.87 | hsa-miR-142-5p | 0.003 |
| hsa-let-7c | 5.18 | hsa-miR-15b | 0.005 |
| hsa-let-7e | 5.18 | hsa-miR-16 | 0.001 |
| hsa-let-7f | 4.79 | hsa-miR-193a-5p | 0.004 |
| hsa-let-7g | 6.14 | hsa-miR-27b* | 0.003 |
| hsa-let-7i | 5.98 | hsa-miR-29b | 0.001 |
| hsa-miR-1 | 2.01 | hsa-miR-30a* | 0.005 |
| hsa-miR-100 | 443.42 | hsa-miR-330-3p | 0.002 |
| hsa-miR-101 | 3.2 | hsa-miR-451 | 0.003 |
| hsa-miR-103 | 5.5 | hsa-miR-452 | 0.001 |
| hsa-miR-107 | 6.17 | hsa-miR-486-5p | 0.191 |
| hsa-miR-10a | 9.28 | hsa-miR-495 | 0.003 |
| hsa-miR-10b | 10.41 | hsa-miR-503 | 0.003 |
| hsa-miR-122 | 2.69 | hsa-miR-518e* | 0.005 |
| hsa-miR-124 | 709.48 | hsa-miR-519a* | 0.005 |
| hsa-miR-125b | 798.16 | hsa-miR-519b-5p | 0.005 |
| hsa-miR-127-3p | 443.42 | hsa-miR-519c-5p | 0.005 |
| hsa-miR-128 | 3.37 | hsa-miR-522* | 0.005 |
| hsa-miR-1283 | 399.08 | hsa-miR-523* | 0.005 |
| hsa-miR-1301 | 532.11 | hsa-miR-886-5p | 0.003 |
| hsa-miR-1308 | 10.12 | hsa-miR-889 | 0.003 |
| hsa-miR-1323 | 16.57 |  |  |
| hsa-miR-140-3p | 2.86 |  |  |
| hsa-miR-143 | 3.9 |  |  |
| hsa-miR-145 | 1241.59 |  |  |
| hsa-miR-151-5p | 310.4 |  |  |
| hsa-miR-181a | 1906.72 |  |  |
| hsa-miR-191 | 3.75 |  |  |
| hsa-miR-192 | 2.6 |  |  |
| hsa-miR-193a-3p | 310.4 |  |  |
| hsa-miR-194 | 620.79 |  |  |
| hsa-miR-195 | 2.84 |  |  |
| hsa-miR-196b | 1285.93 |  |  |
| hsa-miR-1974 | 665.14 |  |  |
| hsa-miR-199a-3p | 15.8 |  |  |
| hsa-miR-199a-5p | 1729.35 |  |  |
| hsa-miR-199b-3p | 15.8 |  |  |
| hsa-miR-200a | 665.14 |  |  |
| hsa-miR-202* | 38.81 |  |  |
| hsa-miR-203 | 2.81 |  |  |
| hsa-miR-204 | 487.77 |  |  |
| hsa-miR-206 | 3.99 |  |  |
| hsa-miR-21 | 9.03 |  |  |
| hsa-miR-2110 | 133.03 |  |  |
| hsa-miR-215 | 798.16 |  |  |
| hsa-miR-22 | 7.45 |  |  |
| hsa-miR-221 | 13.16 |  |  |
| hsa-miR-221* | 620.79 |  |  |
| hsa-miR-222 | 3902.13 |  |  |
| hsa-miR-23a | 10.03 |  |  |
| hsa-miR-23b | 310.4 |  |  |
| hsa-miR-24 | 15.79 |  |  |
| hsa-miR-25 | 443.42 |  |  |
| hsa-miR-26a | 23.91 |  |  |
| hsa-miR-26b | 5.06 |  |  |
| hsa-miR-296-5p | 399.08 |  |  |
| hsa-miR-29a | 5.82 |  |  |
| hsa-miR-29c | 665.14 |  |  |
| hsa-miR-30a | 10.66 |  |  |
| hsa-miR-30c | 399.08 |  |  |
| hsa-miR-30c-1* | 443.42 |  |  |
| hsa-miR-30d | 3.03 |  |  |
| hsa-miR-30e | 4.05 |  |  |
| hsa-miR-31 | 487.77 |  |  |
| hsa-miR-320a | 2.37 |  |  |
| hsa-miR-320b | 2.93 |  |  |
| hsa-miR-320c | 16.87 |  |  |
| hsa-miR-320d | 16.87 |  |  |
| hsa-miR-340 | 2.64 |  |  |
| hsa-miR-34c-5p | 6.12 |  |  |
| hsa-miR-376c | 487.77 |  |  |
| hsa-miR-424 | 6.99 |  |  |
| hsa-miR-432 | 665.14 |  |  |
| hsa-miR-433 | 133.03 |  |  |
| hsa-miR-516a-5p | 532.11 |  |  |
| hsa-miR-516b | 6340.96 |  |  |
| hsa-miR-520d-5p | 399.08 |  |  |
| hsa-miR-9* | 443.42 |  |  |
| hsa-miR-99a | 52.87 |  |  |
| hsa-miR-99b | 2438.83 |  |  |
