# Supplementary material for: Identification of Serum microRNA Biomarkers for Tuberculosis Using RNA-seq
Source: PLoS One. 2014 Feb 20;9(2):e88909. doi: 10.1371/journal.pone.0088909 (PMC3930592; doi:10.1371/journal.pone.0088909)
Supplement: Table S4 — Fold changes in the expression of microRNAs in serum from patients with active TB compared with BCG un-inoculated individuals. (DOC) [file pone.0088909.s004.doc]

**Table S4 Fold changes in the expression of microRNAs in serum from patients with active TB compared with un-inoculated individuals**

| Up-regulated microRNAs | Fold change | Down-regulated microRNAs | Fold change |
| --- | --- | --- | --- |
| hsa-miR-100 | 443.42 | hsa-let-7a | 0.3735 |
| hsa-miR-107 | 1.22 | hsa-let-7d | 0.1393 |
| hsa-miR-10a | 3.28 | hsa-let-7f | 0.1557 |
| hsa-miR-10b | 3.73 | hsa-let-7g | 0.2163 |
| hsa-miR-124 | 2.19 | hsa-let-7i | 0.488 |
| hsa-miR-127-3p | 2.74 | hsa-miR-1 | 0.2345 |
| hsa-miR-128 | 2.52 | hsa-miR-101 | 0.4073 |
| hsa-miR-1283 | 399.08 | hsa-miR-122 | 0.2796 |
| hsa-miR-1323 | 12.18 | hsa-miR-1246 | 0.0031 |
| hsa-miR-140-3p | 1.56 | hsa-miR-126* | 0.0282 |
| hsa-miR-143 | 1.71 | hsa-miR-134 | 0.0021 |
| hsa-miR-145 | 6.71 | hsa-miR-139-3p | 0.0036 |
| hsa-miR-151-5p | 6.71 | hsa-miR-144* | 0.0036 |
| hsa-miR-181a | 4.58 | hsa-miR-146a | 0.0008 |
| hsa-miR-193a-3p | 310.4 | hsa-miR-148a | 0.0036 |
| hsa-miR-194 | 2.68 | hsa-miR-151-3p | 0.0025 |
| hsa-miR-195 | 1418.96 | hsa-miR-152 | 0.0039 |
| hsa-miR-196b | 1285.93 | hsa-miR-15a | 0.0002 |
| hsa-miR-199a-3p | 1.22 | hsa-miR-15b | 0.0009 |
| hsa-miR-199a-5p | 1729.35 | hsa-miR-16 | 0.0002 |
| hsa-miR-199b-3p | 1.22 | hsa-miR-184 | 0.0014 |
| hsa-miR-200a | 2.21 | hsa-miR-185 | 0.0922 |
| hsa-miR-200c | 487.77 | hsa-miR-192 | 0.4199 |
| hsa-miR-202* | 2.52 | hsa-miR-193a-5p | 0.0054 |
| hsa-miR-203 | 443.42 | hsa-miR-193b* | 0.0043 |
| hsa-miR-204 | 487.77 | hsa-miR-221 | 0.4568 |
| hsa-miR-206 | 16.61 | hsa-miR-223* | 0.0008 |
| hsa-miR-21 | 1.18 | hsa-miR-23b* | 0.0017 |
| hsa-miR-22 | 9.24 | hsa-miR-26b | 0.1469 |
| hsa-miR-23a | 4.36 | hsa-miR-29b | 0.0039 |
| hsa-miR-26a | 1.61 | hsa-miR-30b* | 0.0012 |
| hsa-miR-27a | 2.37 | hsa-miR-30e | 0.3334 |
| hsa-miR-296-5p | 399.08 | hsa-miR-30e* | 0.0054 |
| hsa-miR-29a | 2.98 | hsa-miR-32 | 0.0048 |
| hsa-miR-30a | 3.88 | hsa-miR-320a | 0.1527 |
| hsa-miR-30c | 399.08 | hsa-miR-320b | 0.1271 |
| hsa-miR-374b | 443.42 | hsa-miR-320c | 0.0478 |
| hsa-miR-376c | 487.77 | hsa-miR-320d | 0.135 |
| hsa-miR-516a-5p | 532.11 | hsa-miR-330-3p | 0.0054 |
| hsa-miR-516b | 6340.96 | hsa-miR-33a | 0.0014 |
| hsa-miR-520d-5p | 399.08 | hsa-miR-34b* | 0.0027 |
| hsa-miR-9 | 221.71 | hsa-miR-34c-5p | 0.4243 |
| hsa-miR-9* | 443.42 | hsa-miR-378 | 0.1307 |
| hsa-miR-99b | 8.79 | hsa-miR-423-5p | 0.0227 |
|  |  | hsa-miR-451 | 0.0004 |
|  |  | hsa-miR-452 | 0.0004 |
|  |  | hsa-miR-454 | 0.0031 |
|  |  | hsa-miR-483-5p | 0.0043 |
|  |  | hsa-miR-485-5p | 0.002 |
|  |  | hsa-miR-486-5p | 0.0569 |
|  |  | hsa-miR-495 | 0.0031 |
|  |  | hsa-miR-503 | 0.0016 |
|  |  | hsa-miR-522 | 0.0021 |
|  |  | hsa-miR-532-5p | 0.0054 |
|  |  | hsa-miR-548c-5p | 0.0033 |
|  |  | hsa-miR-598 | 0.0031 |
|  |  | hsa-miR-625 | 0.0043 |
|  |  | hsa-miR-652 | 0.0022 |
|  |  | hsa-miR-664* | 0.0025 |
|  |  | hsa-miR-877 | 0.0015 |
|  |  | hsa-miR-889 | 0.0036 |
|  |  | hsa-miR-92a | 0.2834 |
|  |  | hsa-miR-92b | 0.0025 |
|  |  | hsa-miR-93 | 0.0871 |
|  |  | hsa-miR-98 | 0.0003 |
