# Supplementary material for: Identification of Serum microRNA Biomarkers for Tuberculosis Using RNA-seq
Source: PLoS One. 2014 Feb 20;9(2):e88909. doi: 10.1371/journal.pone.0088909 (PMC3930592; doi:10.1371/journal.pone.0088909)
Supplement: Table S5 — Fold changes in the expression of microRNAs in serum from individuals with LTBI compared with BCG-inoculated individuals. (DOC) [file pone.0088909.s005.doc]

**Table S5 Fold changes in the expression of microRNAs in serum from individuals with LTBI compared with BCG-inoculated individuals.**

| Up-regulated microRNAs | Fold change | Down-regulated microRNAs | Fold change |
| --- | --- | --- | --- |
| hsa-let-7a | 2.4 | hsa-miR-1307 | 0.006 |
| hsa-let-7b | 2.5 | hsa-miR-146a | 0.008 |
| hsa-let-7c | 2.05 | hsa-miR-192 | 0.418 |
| hsa-let-7e* | 204.55 | hsa-miR-193a-5p | 0.004 |
| hsa-let-7f | 2.46 | hsa-miR-200c | 0.002 |
| hsa-let-7g | 2.73 | hsa-miR-27b* | 0.003 |
| hsa-let-7i | 3.13 | hsa-miR-330-3p | 0.002 |
| hsa-miR-100 | 715.92 | hsa-miR-340 | 0.365 |
| hsa-miR-103 | 2.3 | hsa-miR-452 | 0.001 |
| hsa-miR-10a | 2.43 | hsa-miR-495 | 0.003 |
| hsa-miR-124 | 409.1 | hsa-miR-886-5p | 0.003 |
| hsa-miR-1301 | 306.82 |  |  |
| hsa-miR-130b | 485.8 |  |  |
| hsa-miR-1323 | 10.7 |  |  |
| hsa-miR-143 | 2.8 |  |  |
| hsa-miR-143* | 511.37 |  |  |
| hsa-miR-144 | 281.25 |  |  |
| hsa-miR-148a | 536.94 |  |  |
| hsa-miR-155 | 255.69 |  |  |
| hsa-miR-15a | 664.78 |  |  |
| hsa-miR-181a | 1048.31 |  |  |
| hsa-miR-182 | 153.41 |  |  |
| hsa-miR-185 | 2.37 |  |  |
| hsa-miR-18a | 281.25 |  |  |
| hsa-miR-191 | 4.32 |  |  |
| hsa-miR-193a-3p | 127.84 |  |  |
| hsa-miR-199a-3p | 7.52 |  |  |
| hsa-miR-199a-5p | 255.69 |  |  |
| hsa-miR-199b-3p | 7.52 |  |  |
| hsa-miR-199b-5p | 153.41 |  |  |
| hsa-miR-202* | 6.81 |  |  |
| hsa-miR-21 | 3.5 |  |  |
| hsa-miR-22 | 2.12 |  |  |
| hsa-miR-221 | 3.18 |  |  |
| hsa-miR-221* | 332.39 |  |  |
| hsa-miR-222 | 2454.58 |  |  |
| hsa-miR-23a | 2.27 |  |  |
| hsa-miR-23b | 255.69 |  |  |
| hsa-miR-23b* | 6.49 |  |  |
| hsa-miR-24 | 5 |  |  |
| hsa-miR-25 | 332.39 |  |  |
| hsa-miR-26a | 4.62 |  |  |
| hsa-miR-26b | 7.09 |  |  |
| hsa-miR-27a | 4.16 |  |  |
| hsa-miR-27b | 2.83 |  |  |
| hsa-miR-28-3p | 178.98 |  |  |
| hsa-miR-29a | 3.17 |  |  |
| hsa-miR-30a | 2.37 |  |  |
| hsa-miR-30e | 3.31 |  |  |
| hsa-miR-320c | 9.73 |  |  |
| hsa-miR-320d | 7.78 |  |  |
| hsa-miR-342-3p | 357.96 |  |  |
| hsa-miR-34c-5p | 2.92 |  |  |
| hsa-miR-375 | 281.25 |  |  |
| hsa-miR-411 | 613.65 |  |  |
| hsa-miR-432 | 230.12 |  |  |
| hsa-miR-455-3p | 127.84 |  |  |
| hsa-miR-483-5p | 664.78 |  |  |
| hsa-miR-487b | 332.39 |  |  |
| hsa-miR-516a-5p | 306.82 |  |  |
| hsa-miR-516b | 843.76 |  |  |
| hsa-miR-517a | 255.69 |  |  |
| hsa-miR-517b | 255.69 |  |  |
| hsa-miR-543 | 204.55 |  |  |
| hsa-miR-598 | 536.94 |  |  |
| hsa-miR-664* | 153.41 |  |  |
| hsa-miR-675 | 511.37 |  |  |
| hsa-miR-885-3p | 127.84 |  |  |
| hsa-miR-9* | 178.98 |  |  |
| hsa-miR-92a | 2.57 |  |  |
| hsa-miR-92b* | 10.22 |  |  |
| hsa-miR-93 | 383.53 |  |  |
| hsa-miR-98 | 178.98 |  |  |
| hsa-miR-99a | 5.51 |  |  |
| hsa-miR-99b | 485.8 |  |  |
