# Supplementary material for: Identification of Serum microRNA Biomarkers for Tuberculosis Using RNA-seq
Source: PLoS One. 2014 Feb 20;9(2):e88909. doi: 10.1371/journal.pone.0088909 (PMC3930592; doi:10.1371/journal.pone.0088909)
Supplement: Table S6 — Fold changes in the expression of microRNAs in serum from individuals with LTBI compared with BCG un-inoculated individuals. (DOC) [file pone.0088909.s006.doc]

**Table S6 Fold changes in the expression of microRNAs in serum from LTBI compared with un-inoculated individuals.**

| Up-regulated microRNAs | Fold change | Down-regulated microRNAs | Fold change |
| --- | --- | --- | --- |
| hsa-let-7a | 0.1557 | hsa-let-7e* | 204.55 |
| hsa-let-7b | 0.3344 | hsa-miR-100 | 715.92 |
| hsa-let-7c | 0.3525 | hsa-miR-130b | 3 |
| hsa-let-7d | 0.0464 | hsa-miR-1323 | 7.87 |
| hsa-let-7e | 0.1335 | hsa-miR-142-5p | 357.96 |
| hsa-let-7f | 0.08 | hsa-miR-143* | 511.37 |
| hsa-let-7g | 0.0961 | hsa-miR-144 | 281.25 |
| hsa-let-7i | 0.2553 | hsa-miR-155 | 255.69 |
| hsa-miR-1 | 0.1827 | hsa-miR-181a | 2.52 |
| hsa-miR-101 | 0.1758 | hsa-miR-18a | 281.25 |
| hsa-miR-103 | 0.4474 | hsa-miR-195 | 536.94 |
| hsa-miR-107 | 0.3565 | hsa-miR-206 | 4.79 |
| hsa-miR-10b | 0.1745 | hsa-miR-22 | 2.61 |
| hsa-miR-122 | 0.0547 | hsa-miR-27a | 3.06 |
| hsa-miR-1246 | 0.0031 | hsa-miR-28-3p | 178.98 |
| hsa-miR-125a-5p | 0.0031 | hsa-miR-29b | 3.21 |
| hsa-miR-125b | 0.0018 | hsa-miR-30a* | 434.67 |
| hsa-miR-126* | 0.0006 | hsa-miR-342-3p | 357.96 |
| hsa-miR-1307 | 0.0029 | hsa-miR-374b | 281.25 |
| hsa-miR-134 | 0.0021 | hsa-miR-411 | 4.42 |
| hsa-miR-144* | 0.0036 | hsa-miR-483-5p | 2.87 |
| hsa-miR-145 | 0.0054 | hsa-miR-487b | 332.39 |
| hsa-miR-146a | 0.0008 | hsa-miR-516a-5p | 306.82 |
| hsa-miR-151-3p | 0.0025 | hsa-miR-516b | 843.76 |
| hsa-miR-152 | 0.0039 | hsa-miR-517a | 255.69 |
| hsa-miR-15a | 0.1652 | hsa-miR-517b | 255.69 |
| hsa-miR-15b | 0.0677 | hsa-miR-675 | 511.37 |
| hsa-miR-184 | 0.1474 | hsa-miR-9* | 178.98 |
| hsa-miR-185 | 0.3627 | hsa-miR-92b* | 536.94 |
| hsa-miR-192 | 0.0674 | hsa-miR-182 | 153.41 |
| hsa-miR-193a-5p | 0.0054 | hsa-miR-193a-3p | 127.84 |
| hsa-miR-193b* | 0.0043 | hsa-miR-210 | 102.27 |
| hsa-miR-194 | 0.0043 | hsa-miR-30c | 102.27 |
| hsa-miR-1974 | 0.001 | hsa-miR-375 | 3.04 |
| hsa-miR-200a | 0.0033 | hsa-miR-455-3p | 127.84 |
| hsa-miR-202* | 0.4421 | hsa-miR-518e* | 153.41 |
| hsa-miR-21 | 0.459 | hsa-miR-519a* | 153.41 |
| hsa-miR-215 | 0.001 | hsa-miR-519b-5p | 153.41 |
| hsa-miR-221 | 0.1103 | hsa-miR-519c-5p | 153.41 |
| hsa-miR-221* | 0.3421 | hsa-miR-522* | 153.41 |
| hsa-miR-223* | 0.0008 | hsa-miR-523* | 153.41 |
| hsa-miR-24 | 0.3421 | hsa-miR-660 | 102.27 |
| hsa-miR-26a | 0.3104 | hsa-miR-885-3p | 127.84 |
| hsa-miR-26b | 0.2058 | hsa-miR-199a-5p | 255.69 |
| hsa-miR-29c | 0.0013 |  |  |
| hsa-miR-30b* | 0.0012 |  |  |
| hsa-miR-30c-1* | 0.0054 |  |  |
| hsa-miR-30e | 0.2723 |  |  |
| hsa-miR-30e* | 0.0054 |  |  |
| hsa-miR-31 | 0.0031 |  |  |
| hsa-miR-32 | 0.0048 |  |  |
| hsa-miR-320a | 0.0693 |  |  |
| hsa-miR-320b | 0.0558 |  |  |
| hsa-miR-320c | 0.0276 |  |  |
| hsa-miR-320d | 0.0623 |  |  |
| hsa-miR-330-3p | 0.0054 |  |  |
| hsa-miR-33a | 0.0014 |  |  |
| hsa-miR-340 | 0.2287 |  |  |
| hsa-miR-34b* | 0.0027 |  |  |
| hsa-miR-34c-5p | 0.2025 |  |  |
| hsa-miR-378 | 0.0314 |  |  |
| hsa-miR-423-5p | 0.0466 |  |  |
| hsa-miR-424 | 0.0804 |  |  |
| hsa-miR-432 | 0.2689 |  |  |
| hsa-miR-451 | 0.2869 |  |  |
| hsa-miR-452 | 0.0004 |  |  |
| hsa-miR-454 | 0.0031 |  |  |
| hsa-miR-485-5p | 0.002 |  |  |
| hsa-miR-486-5p | 0.2435 |  |  |
| hsa-miR-495 | 0.0031 |  |  |
| hsa-miR-522 | 0.0021 |  |  |
| hsa-miR-532-5p | 0.0054 |  |  |
| hsa-miR-548c-5p | 0.0033 |  |  |
| hsa-miR-652 | 0.0022 |  |  |
| hsa-miR-744 | 0.1551 |  |  |
| hsa-miR-877 | 0.0015 |  |  |
| hsa-miR-92b | 0.0025 |  |  |
| hsa-miR-98 | 0.0484 |  |  |
| hsa-miR-99a | 0.087 |  |  |
| hsa-let-7d* | 0.0062 |  |  |
| hsa-miR-1255b | 0.0062 |  |  |
| hsa-miR-127-3p | 0.0062 |  |  |
| hsa-miR-1277 | 0.0086 |  |  |
| hsa-miR-141 | 0.0072 |  |  |
| hsa-miR-148b | 0.0086 |  |  |
| hsa-miR-187 | 0.0086 |  |  |
| hsa-miR-223 | 0.0086 |  |  |
| hsa-miR-363* | 0.0086 |  |  |
| hsa-miR-374a* | 0.0062 |  |  |
| hsa-miR-423-3p | 0.0062 |  |  |
| hsa-miR-499-5p | 0.0072 |  |  |
| hsa-miR-548j | 0.0087 |  |  |
| hsa-miR-664* | 0.3901 |  |  |
| hsa-miR-760 | 0.0072 |  |  |
