# Supplementary material for: Identification of Serum microRNA Biomarkers for Tuberculosis Using RNA-seq
Source: PLoS One. 2014 Feb 20;9(2):e88909. doi: 10.1371/journal.pone.0088909 (PMC3930592; doi:10.1371/journal.pone.0088909)
Supplement: Table S7 — Fold changes in the expression of microRNAs in serum from BCG-inoculated compared with BCG un-inoculated individuals. (DOC) [file pone.0088909.s007.doc]

**Table S7 Fold changes in the expression of microRNAs in serum from BCG-inoculated compared with un-inoculated individuals**

| Up-regulated microRNAs | Fold change | Down-regulated microRNAs | Fold change |
| --- | --- | --- | --- |
| hsa-miR-195 | 499.29 | hsa-let-7a | 0.0649 |
| hsa-miR-200c | 499.29 | hsa-let-7b | 0.1340 |
| hsa-miR-374b | 473.02 | hsa-let-7c | 0.1720 |
| hsa-miR-142-5p | 367.9 | hsa-let-7d | 0.0718 |
| hsa-miR-27b* | 315.34 | hsa-let-7d* | 0.0062 |
| hsa-miR-30a* | 210.23 | hsa-let-7e | 0.1004 |
| hsa-miR-518e* | 183.95 | hsa-let-7f | 0.0325 |
| hsa-miR-519a* | 183.95 | hsa-let-7g | 0.0353 |
| hsa-miR-519b-5p | 183.95 | hsa-let-7i | 0.0817 |
| hsa-miR-519c-5p | 183.95 | hsa-miR-1 | 0.1164 |
| hsa-miR-522* | 183.95 | hsa-miR-101 | 0.1272 |
| hsa-miR-523* | 183.95 | hsa-miR-103 | 0.1945 |
| hsa-miR-203 | 157.67 | hsa-miR-107 | 0.1979 |
| hsa-miR-1979 | 105.11 | hsa-miR-10a | 0.3534 |
| hsa-miR-886-5p | 6.25 | hsa-miR-10b | 0.3587 |
| hsa-miR-206 | 4.17 | hsa-miR-122 | 0.1040 |
| hsa-miR-330-3p | 2.98 | hsa-miR-124 | 0.0031 |
| hsa-miR-29b | 2.69 | hsa-miR-1246 | 0.0031 |
|  |  | hsa-miR-1255b | 0.0062 |
|  |  | hsa-miR-125a-5p | 0.0031 |
|  |  | hsa-miR-125b | 0.0018 |
|  |  | hsa-miR-126* | 0.0006 |
|  |  | hsa-miR-127-3p | 0.0062 |
|  |  | hsa-miR-1277 | 0.0086 |
|  |  | hsa-miR-1301 | 0.0018 |
|  |  | hsa-miR-1308 | 0.1033 |
|  |  | hsa-miR-130b | 0.0062 |
|  |  | hsa-miR-134 | 0.0021 |
|  |  | hsa-miR-141 | 0.0072 |
|  |  | hsa-miR-143 | 0.4374 |
|  |  | hsa-miR-144* | 0.0036 |
|  |  | hsa-miR-145 | 0.0054 |
|  |  | hsa-miR-146a | 0.1014 |
|  |  | hsa-miR-148a | 0.0036 |
|  |  | hsa-miR-148b | 0.0086 |
|  |  | hsa-miR-151-3p | 0.0025 |
|  |  | hsa-miR-152 | 0.0039 |
|  |  | hsa-miR-15a | 0.0002 |
|  |  | hsa-miR-15b | 0.1623 |
|  |  | hsa-miR-16 | 0.3715 |
|  |  | hsa-miR-181a | 0.0024 |
|  |  | hsa-miR-184 | 0.0014 |
|  |  | hsa-miR-185 | 0.1528 |
|  |  | hsa-miR-187 | 0.0086 |
|  |  | hsa-miR-191 | 0.1690 |
|  |  | hsa-miR-192 | 0.1613 |
|  |  | hsa-miR-193b* | 0.0043 |
|  |  | hsa-miR-194 | 0.0043 |
|  |  | hsa-miR-1974 | 0.001 |
|  |  | hsa-miR-199a-3p | 0.0771 |
|  |  | hsa-miR-199b-3p | 0.0771 |
|  |  | hsa-miR-199b-5p | 0.0072 |
|  |  | hsa-miR-200a | 0.0033 |
|  |  | hsa-miR-202* | 0.0649 |
|  |  | hsa-miR-21 | 0.1310 |
|  |  | hsa-miR-2110 | 0.0054 |
|  |  | hsa-miR-215 | 0.0009 |
|  |  | hsa-miR-221 | 0.0347 |
|  |  | hsa-miR-221* | 0.0011 |
|  |  | hsa-miR-222 | 0.0002 |
|  |  | hsa-miR-223* | 0.0008 |
|  |  | hsa-miR-23a | 0.4351 |
|  |  | hsa-miR-23b | 0.0033 |
|  |  | hsa-miR-23b* | 0.1363 |
|  |  | hsa-miR-24 | 0.0683 |
|  |  | hsa-miR-25 | 0.0020 |
|  |  | hsa-miR-26a | 0.0672 |
|  |  | hsa-miR-26b | 0.0290 |
|  |  | hsa-miR-29c | 0.0013 |
|  |  | hsa-miR-30a | 0.3643 |
|  |  | hsa-miR-30b* | 0.0012 |
|  |  | hsa-miR-30c-1* | 0.0054 |
|  |  | hsa-miR-30d | 0.3310 |
|  |  | hsa-miR-30e | 0.0823 |
|  |  | hsa-miR-30e* | 0.0054 |
|  |  | hsa-miR-31 | 0.0031 |
|  |  | hsa-miR-32 | 0.0048 |
|  |  | hsa-miR-320a | 0.0643 |
|  |  | hsa-miR-320b | 0.0433 |
|  |  | hsa-miR-320c | 0.0028 |
|  |  | hsa-miR-320d | 0.0080 |
|  |  | hsa-miR-33a | 0.0014 |
|  |  | hsa-miR-34b* | 0.0027 |
|  |  | hsa-miR-34c-5p | 0.0694 |
|  |  | hsa-miR-363* | 0.00864 |
|  |  | hsa-miR-374a* | 0.0062 |
|  |  | hsa-miR-378 | 0.0775 |
|  |  | hsa-miR-411 | 0.0072 |
|  |  | hsa-miR-423-5p | 0.0425 |
|  |  | hsa-miR-424 | 0.1446 |
|  |  | hsa-miR-432 | 0.0012 |
|  |  | hsa-miR-451 | 0.1529 |
|  |  | hsa-miR-452 | 0.3322 |
|  |  | hsa-miR-454 | 0.0031 |
|  |  | hsa-miR-483-5p | 0.0043 |
|  |  | hsa-miR-485-5p | 0.0020 |
|  |  | hsa-miR-486-5p | 0.2984 |
|  |  | hsa-miR-499-5p | 0.0072 |
|  |  | hsa-miR-503 | 0.5049 |
|  |  | hsa-miR-522 | 0.0021 |
|  |  | hsa-miR-532-5p | 0.0054 |
|  |  | hsa-miR-543 | 0.0072 |
|  |  | hsa-miR-548c-5p | 0.0033 |
|  |  | hsa-miR-548j | 0.0086 |
|  |  | hsa-miR-598 | 0.0031 |
|  |  | hsa-miR-652 | 0.0022 |
|  |  | hsa-miR-664* | 0.0025 |
|  |  | hsa-miR-744 | 0.2591 |
|  |  | hsa-miR-760 | 0.0072 |
|  |  | hsa-miR-877 | 0.0015 |
|  |  | hsa-miR-92a | 0.2249 |
|  |  | hsa-miR-92b | 0.2673 |
|  |  | hsa-miR-93 | 0.0020 |
|  |  | hsa-miR-98 | 0.0003 |
|  |  | hsa-miR-99a | 0.01578 |
|  |  | hsa-miR-99b | 0.0036 |
