# Supplementary material for: Identification of Serum microRNA Biomarkers for Tuberculosis Using RNA-seq
Source: PLoS One. 2014 Feb 20;9(2):e88909. doi: 10.1371/journal.pone.0088909 (PMC3930592; doi:10.1371/journal.pone.0088909)
Supplement: Table S8 — Pathways represented among the genes predicted to be targeted by microRNAs differentially-expressed in serum from patients with active TB compared with the other controls (LTBI, BCG-inoculated and un-inoculated individuals). (DOC) [file pone.0088909.s008.doc]

**Table S8 KEGG pathways represented among the genes predicted to be targeted by microRNAs differentially-expressed in serum from patients with active TB compared with the other controls (LTBI, BCG-inoculated and un-inoculated individuals)**

| Pathway Name | P-value |
| --- | --- |
| Pathways in cancer | 6.20E-18 |
| Focal adhesion | 3.20E-16 |
| Wnt signaling pathway | 5.20E-12 |
| Endocytosis | 8.20E-12 |
| Axon guidance | 1.30E-11 |
| Renal cell carcinoma | 4.10E-11 |
| MAPK signaling pathway | 1.30E-10 |
| ErbB signaling pathway | 1.40E-10 |
| Chronic myeloid leukemia | 1.40E-09 |
| Colorectal cancer | 1.40E-09 |
| Neurotrophin signaling pathway | 4.10E-09 |
| Prostate cancer | 6.60E-09 |
| Glioma | 5.90E-08 |
| Adherens junction | 6.90E-08 |
| ECM-receptor interaction | 6.90E-07 |
| Insulin signaling pathway | 1.90E-06 |
| Regulation of actin cytoskeleton | 2.60E-06 |
| Non-small cell lung cancer | 3.60E-06 |
| Long-term potentiation | 1.00E-05 |
| Melanogenesis | 1.40E-05 |
| Small cell lung cancer | 1.60E-05 |
| Pancreatic cancer | 2.00E-05 |
| Fc gamma R-mediated phagocytosis | 2.10E-05 |
| Acute myeloid leukemia | 2.80E-05 |
| Melanoma | 3.70E-05 |
| Ubiquitin mediated proteolysis | 8.00E-05 |
| GnRH signaling pathway | 1.30E-04 |
| Dilated cardiomyopathy | 2.40E-04 |
| TGF-beta signaling pathway | 2.70E-04 |
| Tight junction | 3.00E-04 |
| Adipocytokine signaling pathway | 3.90E-04 |
| Endometrial cancer | 4.50E-04 |
| T cell receptor signaling pathway | 4.70E-04 |
| Arrhythmogenic right ventricular cardiomyopathy (ARVC) | 5.60E-04 |
| Hypertrophic cardiomyopathy (HCM) | 7.40E-04 |
| Gap junction | 1.10E-03 |
| mTOR signaling pathway | 1.20E-03 |
| Thyroid cancer | 2.90E-03 |
| Phosphatidylinositol signaling system | 3.30E-03 |
| Basal cell carcinoma | 3.30E-03 |
| Hedgehog signaling pathway | 4.50E-03 |
| SNARE interactions in vesicular transport | 5.30E-03 |
| Aldosterone-regulated sodium reabsorption | 6.10E-03 |
| Notch signaling pathway | 7.60E-03 |
| Progesterone-mediated oocyte maturation | 7.70E-03 |
| VEGF signaling pathway | 8.40E-03 |
| B cell receptor signaling pathway | 8.40E-03 |
| Amyotrophic lateral sclerosis (ALS) | 9.00E-03 |
| Oocyte meiosis | 9.10E-03 |
